# Incidence and groups at risk for unexpected uterine leiomyosarcoma: a Dutch nationwide cohort study

**DOI:** 10.1007/s00404-018-4949-4

**Published:** 2018-11-30

**Authors:** Lukas van den Haak, Cor. D. de Kroon, Milo. I. Warmerdam, Albert G. Siebers, Johann P. Rhemrev, Theodoor. E. Nieboer, Frank Willem Jansen

**Affiliations:** 1 Department of Gynecology, Leiden University Medical Center, PO Box 9600, 2300 RC Leiden, The Netherlands; 2 The Nationwide Network and Registry of Histo- and Cytopathology in The Netherlands, Utrecht, The Netherlands; 3 Department of Obstetrics and Gynecology, Haaglanden Medisch Centrum, The Hague, The Netherlands; 4 Department of Obstetrics and Gynecology, Radboud University Medical Center, Nijmegen, The Netherlands; 5 Department BioMechanical Engineering, Delft University of Technology, 2628 CD Delft, The Netherlands

**Keywords:** Hysterectomy, Laparoscopy, Leiomyosarcoma, Morcellation

## Abstract

**Objective:**

To estimate the risk of uterine leiomyosarcoma in patients undergoing gynecological surgery and also to identify groups at risk for unrecognized uterine leiomyosarcoma.

**Methods:**

A national cohort study was performed evaluating all uterine leiomyosarcoma (ULMS) diagnosed in The Netherlands between January 2000 and September 2015. Cases were identified and supplied by the nationwide network and registry of histo- and cytopathology in The Netherlands (PALGA). Unexpected and expected ULMS were compared. Approval for this study was granted by the Medical Ethics Committee of all participating hospitals and by the review board of PALGA.

**Results:**

262 original cases were included. The overall incidence of ULMS in our study was 0.25% or 1:400 patients. The incidence of unexpected ULMS was 0.12% or 1:865 patients. Preoperatively, a malignancy was unexpected in 46% of the cases and expected in 54%. Abnormal uterine bleeding constituted most of the symptoms. 90% of women underwent abdominal hysterectomy and/or bilateral salpingo-oophorectomy.

**Conclusions:**

Leiomyosarcoma are rare. Women aged 40–50 years with abnormal uterine bleeding are most at risk for unexpected ULMS. In contrast, this risk is low in postmenopausal women. ULMS were highly uncommon in women aged under 40 years.

## Introduction

The number of laparoscopic procedures has decreased in favor of laparotomy, since the Food and Drug Administrations (FDA) decided to discourage power morcellation [1–5] This decision was based on the occurrence of unexpected uterine (leiomyo)sarcoma during hysterectomy or myomectomy for presumed benign fibroids. It was calculated by the FDA that this risk is as high as 1 in 498 for uterine leiomyosarcoma (ULMS) [6]. However, the evidence that formed the basis for this calculation has been criticized for its weakness and potential bias. For instance, mainly single-center studies were used and preoperatively diagnosed malignancies were included [7, 8]. Recently, the FDA has updated this risk of occult ULMS to 1 in 495 to 1 in 1100 women undergoing surgery, using data from more recent studies [9]. Applying this notable range to a decision analysis for perioperative risk estimations regarding laparoscopic hysterectomy versus laparotomy, scenarios can be found in favor for both approaches [10]. To improve the accuracy of such models and thus better inform patients, more data on the actual incidence of (unexpected) ULMS are needed. The primary aim of our study was to expand the current data by calculating the risk of unexpected ULMS during gynecological procedures in The Netherlands. Secondly, we attempted to identify groups at relatively high or low risk for ULMS to enhance the preoperative selection for the proper surgical procedure of these patients.

## Methods and materials

Approval for this study was granted by the Medical Ethics Committee of all participating hospitals and by the review board of PALGA.

A national cohort study was performed evaluating all patients diagnosed with ULMS in The Netherlands between January 2000 and September 2015. Cases were identified and supplied by the nationwide network and registry of histo- and cytopathology in The Netherlands (PALGA) [11]. Women with a histo-pathologically confirmed ULMS diagnosis after surgical treatment (abdominal, vaginal and laparoscopic hysterectomy; hysteroscopic, laparoscopic and abdominal myomectomy; staging laparotomy and debulking surgery) were included. Only the initial procedure identifying the ULMS was considered, to avoid multiple registration of the same case. This naturally implies that second opinions of these cases, although registered in the PALGA database, were excluded. Basic patient characteristics, relevant medical history, clinical presentation and the preoperative diagnostics were retrieved from medical charts. All abnormal bleeding patterns (including excessive, irregulair or postmenopausal) were defined as abnormal uterine bleeding. Size of myoma was measured in centimeters or compared to weeks of gestation. Rapid growth of myoma was considered present if this was explicitly stated in the medical charts. Cases were classified as unexpected ULMS if (any type of) malignancy was not considered preoperatively, was not stated as indication for surgery, or if surgical techniques were used that were not in accordance with ULMS treatment guidelines (meaning abdominal hysterectomy, with or without salpingo-oophorectomy). Preoperative ultrasound (US), computed tomography (CT), magnetic resonance imaging (MRI), hysteroscopy and endometrial sampling/curettage were considered suspicious if (any type of) malignancy was considered by the examining gynecologist, radiologist or pathologist. To calculate the risk of ULMS in surgical specimens in our cohort, the number of all types of benign tumors of the myometrium was used during the same inclusion period. This number was also derived from the PALGA database and consisted of leiomyoma (epithelioid, myxoid, cellular, bizar, angioleiomyoma, angiomyoleiomoma), angiomyofibroblastoma and inflammatory pseudotumors. An independent student t test, a Pearson Chi square test and Fisher exact test were used where applicable. Differences with a *p* value < 0.05 were considered statistically significant. SPSS 20 was used to analyze all data.

To compare our data, a literature search was performed using the PubMed, Web of Science, Embase and Cochrane databases. Search terms consisted of ‘hysterectomy’, ‘myomectomy’, ‘uterine (leiomyo)sarcoma’, ‘risk’, ‘prevalence’ and ‘incidence’. Only original cohorts from multicenter studies evaluating ULMS were included to match our cohort as well as possible.

## Results

**Fig. 1:**
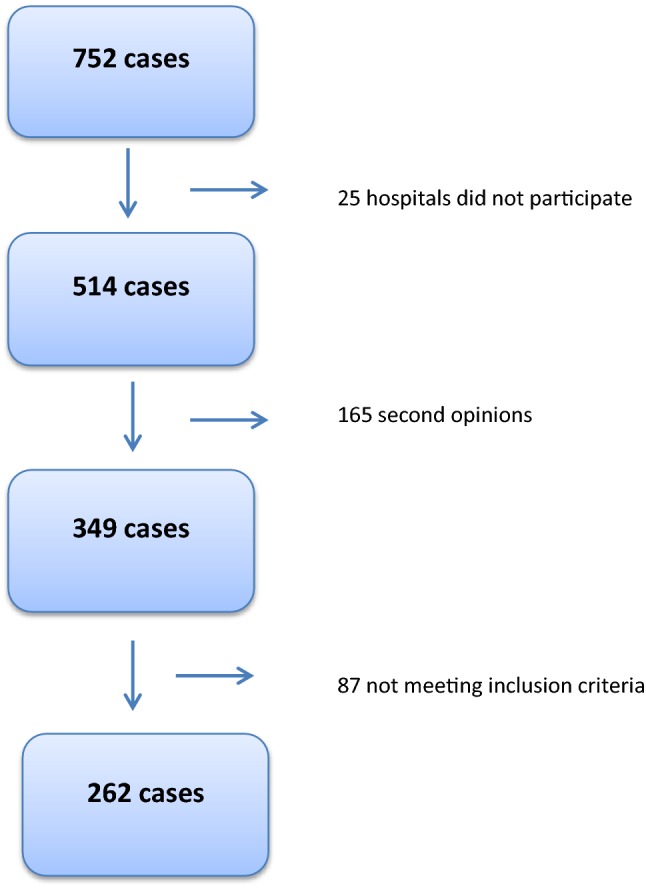
Inclusion flowchart. 25 hospitals did not participate in the majority of instances without reason. Second opinions consisted of double registrations in the PALGA system. Only the first original case was included in this study. Not meeting inclusion criteria: 14 stromal tumors of unknown significance (STUMP), 5 endometrial stromal sarcoma (ESS), 4 carcinosarcoma, 2 adenosarcoma, 1 malignant mixed müllarian tumor, 1 undifferentiated endometrial carcinoma, 2 cellular leiomyoma, 43 other reasons (non-gynecological sarcomatoid tumors or recurrences of primary tumors not eligible for inclusion), and from 15 cases no chart could be found

From January 2000 until September 2015, 752 ULMS were registered in The Netherlands by the PALGA database, originating from 67 hospitals. 43 hospitals (63%) were willing to participate in this study, comprising 6 academic referral centers (of 8 in total), 2 additional tertiary referral centers (of 2), and 35 general hospitals (of 57). These hospitals reflect 514 cases (72%). 252 cases were excluded because they were not original cases (mainly second opinion referrals to specialized pathology centres to confirm the diagnosis) or due to not meeting the inclusion criteria (Fig. 1). In all, 262 original cases were eligible for inclusion of these, the medical records were missing from 26 cases and only the original pathology report could be found. These cases were, therefore, only used to calculate the risk of ULMS and not for patient characteristics.

**Fig. 2:**
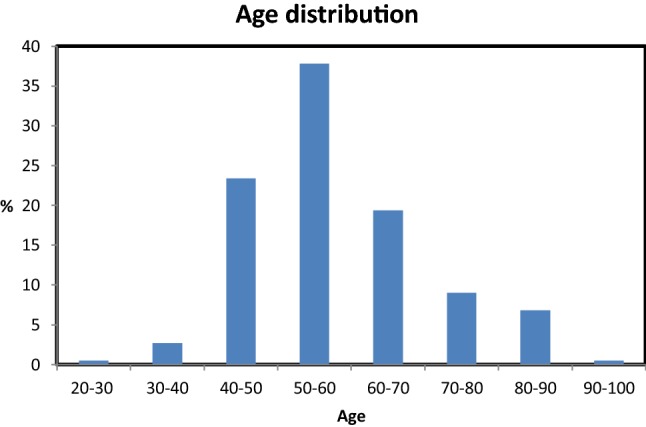
Age distribution of our cohort (%)

Basic characteristics are shown in Table 1. Of the cases of ULMS, 54% were suspected of having a malignancy and 46% were unexpected. The mean age in the expected group was 62 (range 20–91) and it was 52 (range 31–81) in the unexpected group. ULMS was most often found in women aged 50-60 years as is demonstrated by the age distribution in Fig. 2. Sixty-seven percent of the unexpected cases concerned premenopausal women and 17% of the expected cases were premenopausal. Abnormal uterine bleeding (AUB) constituted most of the symptoms: 43% overall and 52% versus 33% in the unexpected and expected group.

**Table 1:** Basic characteristics

	Cohort	Unexpected	Expected
*N*	236	109 (46%)	127 (54%)
Age	58 (12, 20–91)	52 (9, 31–81)*	62 (12, 20–91)*
Menopause			
Pre	40	67*	17*
Post	60	33*	83*
Symptoms			
Pain	15	14	16
AUB	43	52	33
AUB + pain	12	12	12
Mass effect	21	20	22
Weight loss	8	0	15
None	2	2	2
Type of surgery			
AH	25	46	5
AH + BSO	46	30	65
LH	4	7	2
VH	1	3	0
MM	2	4	0
TCRM	4	7	1
Debulking	13	1	26
Other	5	3	1
No of myoma			
One	64	57	72
> One	36	43	28
Uterine size^a^	20	19*	22*
Myoma size^b^	10	9*	12*
Rapid myoma growth			
No	33	44	18
Yes	67	56	82

Age: mean (standard deviation, range); expected/symptoms/type of surgery: percentages. Rapid myoma growth based on 42 cases

*AUB* abnormal uterine bleeding, *AH* abdominal hysterectomy, *BSO* bilateral salpingo-oophorectomy, *LH* laparoscopic hysterectomy, *VH* vaginal hysterectomy, *MM* myomectomy, *TCRM* transcervical resection of myoma

*Significant at *p* ≤ 0.05

^a^Uterine size based on 82 cases

^b^Myoma size based on 139 cases

The preoperative average uterine size was in accordance with 20 weeks of gestation (based on 82 cases) and preoperative average myoma size was 10 cm (based on 139 cases). Uterus and myoma were larger in the expected ULMS group: 19 weeks versus 22 weeks, *p* 0.01 and 9 cm vs 12 cm, *p* 0.003. For the majority of cases (64%), the myoma was solitary (based on 137 cases). In cases with multiple myoma, a malignancy was less often expected: 34%, *p* < 0.06. Rapid myoma growth was reported in 67% of cases (based on 42 cases). No differences were found regarding growth and menopausal status or expected versus unexpected ULMS.

Next, the preoperative workup and treatment are presented. Nearly, all patients (99%) received an US. CT and MRI were performed in 29% and 7% of cases, respectively, hysteroscopy in 16% and sampling of the endometrium in 38%. For US, CT, MRI, hysteroscopy and sampling, respectively, 37, 75, 56, 32 and 45% of the findings were indicative of a malignancy (Table 2). US and sampling were more often suspicious in postmenopausal patients than in premenopausal patients (51% versus 20%, *p* < 0.001 and 58% versus 17%, *p* < 0.001, respectively).

**Table 2:** Preoperative diagnostic workup

	Cohort (*N* = 236)	Premenopausal	Postmenopausal
Ultrasound			
Total	99	98	99
Suspicious	37	20*	51*
CT			
Total	29	19	36
Suspicious	75	71	76
MRI			
Total	7	7	7
Suspicious	56	43	67
Hysteroscopy			
Total	16	13	29
Suspicious	32	18	39
Endometrial Sampling			
Total	38	32	44
Suspicious	45	17*	57*

Numbers are percentages of the cohort

*Significant at *p* ≤ 0.05

Most women (69%) were treated by abdominal hysterectomy with or without bilateral salpingo-oophorectomy (AH ± BSO). An additional 15% of women received staging laparotomy or debulking surgery. Laparoscopic hysterectomy (LH) was used in only in 4% of all women. In the unexpected group, power morcellation was used in 2 cases. In addition, manual morcellation was performed in 2 other cases: to accommodate vaginal extraction of the uterus after LH, and during conversion of vaginal hysterectomy (VH) to AH.

During the same inclusion period as our cohort, 144.431 benign tumors of the myometrium were registered by PALGA. Consequently, the overall incidence of ULMS in our study was 0.25% or 1:400 patients. The risk of unexpected ULMS was 0.12% or 1:865 patients. The risk of receiving other treatment for ULMS than AH ± BSO or staging/debulking in the unexpected group was 0.04% or 1:2500 patients.

## Discussion

This nationwide cohort study evaluated all ULMS cases in The Netherlands from January 2000–September 2015. The risk of encountering an unexpected ULMS was 0.12% or 1:865 patients. Moreover, the risk for patients with ULMS to undergo surgical treatment other than AH ± BSO, staging or debulking was 0.04% or 1:2500 patients. These numbers are in concurrence with the studies found in our literature search. In total, 7 multicenter cohorts were found with incidences ranging from 2.3% or 1:44 to 0.07% or 1:1465 cases [12–18] (Table 3). Unfortunately, a meta-analysis of the data from these studies could not be performed due to heterogeneity of the included study population.

**Table 3:** Multicenter original cohort studies [11–17]

Author	Study	Period	Origin	Population	ULMS risk
Skorstad et al. (2016) [12]	Retrospective nationwide cohort	2000–2012	Cancer Registry of Norway	Women undergoing laparoscopy due to abnormal uterine bleeding or leiomyoma	0.08%/1:1250
Oduyebo et al. (2016) [13]	Retrospective case controlled	Jan 2005–Aug 2012	Brigham and Women’s Hospital and Dana-Farber Cancer Institute	Women undergoing myomectomy or hysterectomy via robot or laparoscopy with electromechanical or manual morcellation	0.19%/1:526
Rodriguez et al. (2016) [14]	Retrospective cohort	2002–2011	Clinformatics DataMart database	Women aged 25-64 with leiomyoma undergoing laparoscopic supracervical hysterectomy or myomectomy	0.14%/1:714
Raine-Bennett et al. (2015) [15]	Retrospective population based cohort	2006–2013	Kaiser Permanente’s electronic health record and regional claims systems	Women over 18 years undergoing hysterectomy for leiomyoma	0.23%/1:429
Raspagliesi et al. (2017) [16]	Retrospective cohort	2004–2014	8 health centers of the MITO group	Women over 18 years undergoing surgery for leiomyoma	2.3%/1:44
Nugent et al. (2015) [18]	Retrospective cohort	2000–2014	German multi-centers group (VAAO) + 2 additional hospitals	Women with AUB, fibroids and/or pain undergoing LSH or LM	0.07%/1:1465
Parker et al. (1994) [17]	Retrospective cohort	1988–1992	Santa Monica Hospital and St. John’s Hospital. California	All women undergoing surgery for leiomyoma	0.08%/1:1250
Current study	Retrospective cohort	Jan 2000–Sept 2015	PALGA nationale database	All women with pathology confirmed ULMS and surgical treatment	0.12%/1:865

Based on our evaluation, certain groups are at higher risk for preoperatively unrecognized ULMS than others. First, women aged 40 years and younger constituted only 4% of our cohort. Therefore, minimally invasive and/or fertility sparing treatments such as a laparoscopic myomectomy could be considered for these women. The highest risk for preoperatively unrecognized ULMS was found in women aged 40–50. In this age group, a malignancy was suspected in only 15% of the women as opposed to 53% and 63% in women aged 50–60 and 60–70, respectively. In women over 70 years of age, a malignancy was suspected in over 80%. Furthermore, symptoms and preoperative workup were not distinctive for this high-risk group. In our cohort, most premenopausal women complained of AUB and, in contrast to postmenopausal women, this usually does not indicate a malignancy. Furthermore, as AUB and fibroids are the main indication for hysterectomy in benign conditions, these women are likely to undergo surgery [19].

Next, a significant difference was found between uterus size and myoma size in unexpected and expected cases. Yet, these differences were small and size was overall large in both groups. Furthermore, these results should be interpreted with caution because possibly only distinctive cases were well registered.

Finally, it was found that preoperative diagnostics were less likely to diagnose a malignancy in our cohort of premenopausal women. For instance, endometrial sampling demonstrated a malignancy in 57% of postmenopausal women compared to only in 17% of premenopausal women. Although US is often a readily available diagnostic test, the diagnostic value in our cohort was low. Interestingly, an evaluation of tumor vascularity and Doppler measurements was not performed but in a few cases, although this could be due to suboptimal reporting and due to the time span of the cohort. These measurements should not be overlooked as meanwhile favorable numbers regarding sensitivity, specificity and positive predictive value for ULMS have been described [20]. The vast majority of CT imaging (89%) was reserved for women over 50 years of age. Naturally, in this group malignancies were more often suspected and CT was used to confirm the suspicion raised by a patients history, or to aid in staging of the disease. However, in light of the aforementioned risk group it is interesting to notice that in women aged 40–50, a CT and MRI was performed in a minority of cases. One explanation may be that these women were previously not considered at risk for ULMS. An increased awareness may thus aid in reducing the number of unexpected ULMS in this group.

Our study has some potential weaknesses. Not all institutes were willing to participate; therefore, not all cases could be verified. Next, due to the retrospective design, missing data occurred. A surprisingly low number of patients were treated by minimally invasive surgical treatments, explaining the very low risk for patients with unexpected ULMS to undergo non-standard oncological treatments. Therefore, this risk (1:2500) may have limited external validity. The strength of our study is the nationwide cohort. Almost all tertiary care academic centers as well as the majority of general care hospitals in The Netherlands participated in this study. In our literature search, only 1 other study encompasses true nationwide data [12]. This study consisted of women undergoing laparoscopy for abnormal uterine bleeding or leiomyoma. Our study evaluated all ULMS cases, eliminating selection bias due to treatment groups. Therefore, notwithstanding the shortcomings, our data are a valuable addition to the already existing evidence. Furthermore, our study identified high- and low-risk groups, thereby offering an additional means in clinical practice to decide a treatment strategy together with the patient. Future studies will include a matched case–control study using this cohort, to further define risk factors for ULMS. However, finding proper matched cases will be challenging. Also, given the increase in laparoscopic procedures in the past decade it will be of interest to analyze a more recent cohort to compare the number of expected versus unexpected cases and the number of patients who received suboptimal surgical treatment.

## Conclusion

The risk of ULMS is overall low and the majority of cases were expected. Women aged 40–50 years with AUB are most at risk for unexpected malignancies. ULMS was highly uncommon in women aged under 40 years.
